# Highly Efficient Transfer of 7TM Membrane Protein from Native Membrane to Covalently Circularized Nanodisc

**DOI:** 10.1038/s41598-018-31925-1

**Published:** 2018-09-10

**Authors:** Vivien Yeh, Tsung-Yen Lee, Chung-Wen Chen, Pai-Chia Kuo, Jessie Shiue, Li-Kang Chu, Tsyr-Yan Yu

**Affiliations:** 1 Institute of Atomic and Molecular Sciences, Academia Sinica, 1, Sec. 4, Roosevelt Rd., Taipei, 10617 Taiwan; 2 Department of Chemistry, National Tsing Hua University, 101, Sec. 2, Kuang-Fu Rd., Hsinchu, 30013 Taiwan; 3 Department of Chemistry, National Taiwan University, 1, Sec. 4, Roosevelt Rd., Taipei, 10617 Taiwan; 4 Institute of Physics, Academia Sinica, No.128, Sec. 2, Academia Rd., Taipei, 11529 Taiwan

## Abstract

Incorporating membrane proteins into membrane mimicking systems is an essential process for biophysical studies and structure determination. Monodisperse lipid nanodiscs have been found to be a suitable tool, as they provide a near-native lipid bilayer environment. Recently, a covalently circularized nanodisc (cND) assembled with a membrane scaffold protein (MSP) in circular form, instead of conventional linear form, has emerged. Covalently circularized nanodiscs have been shown to have improved stability, however the optimal strategies for the incorporation of membrane proteins, as well as the physicochemical properties of the membrane protein embedded in the cND, have not been studied. Bacteriorhodopsin (bR) is a seven-transmembrane helix (7TM) membrane protein, and it forms a two dimensional crystal consisting of trimeric bR on the purple membrane of halophilic archea. Here it is reported that the bR trimer in its active form can be directly incorporated into a cND from its native purple membrane. Furthermore, the assembly conditions of the native purple membrane nanodisc (PMND) were optimized to achieve homogeneity and high yield using a high sodium chloride concentration. Additionally, the native PMND was demonstrated to have the ability to assemble over a range of different pHs, suggesting flexibility in the preparation conditions. The native PMND was then found to not only preserve the trimeric structure of bR and most of the native lipids in the PM, but also maintained the photocycle function of bR. This suggests a promising potential for assembling a cND with a 7TM membrane protein, extracted directly from its native membrane environment, while preserving the protein conformation and lipid composition.

## Introduction

Membrane proteins are estimated to account for 20–30% of the encoded protein in most genomes^1^. Additionally, they are involved in a variety of vital cellular processes such as signal transduction and ion transportation across the membrane^2^. Due to the high hydrophobicity of most membrane proteins, membrane mimics have been used to provide a stable environment for biophysical characterization *in vitro*^3^. Amongst the different membrane mimics^4^, monodisperse lipid nanodiscs have been used as a suitable membrane mimic for both structural and functional studies^5–9^. This is because they provide the necessary lipid bilayer environment with flexibility possible in the lipid composition, while also being highly stable and uniform in size^10^. Nanodiscs have been used to incorporate purified membrane proteins, however this means that the target protein must have been previously isolated and solubilized using detergent prior to the nanodisc assembly. The lipid molecules, which in most cases are commercial synthetic lipids, are then mixed with the detergent solubilized membrane protein and the membrane scaffold protein (MSP) to form the nanodisc. However, the native membrane bilayer and its lipid composition have unique properties that have specific effects on the functions, stability and/or folding of membrane proteins^11,12^. Therefore, despite providing a near-native membrane bilayer, the lipids that make up the nanodisc can still influence the lipid-protein interaction and alter the behavior of the membrane protein^8,13,14^.

Previously it has been established that a purified, detergent solubilized bacterial chemotactic receptor from *Escherichia coli* can be successfully reconstituted into a nanodisc composed of *E*. *coli* lipid extracts^15^. While the application of nanodiscs has been mostly employed to incorporate purified proteins into lipid bilayers, studies have demonstrated the feasibility of encapsulating proteins from host cell membranes with supplemented synthetic lipid molecules^16–18^. The concept of direct assembly of the nanodisc from the microsomal membrane has been demonstrated with overexpressed cytochrome P450 in baculovirus-infected insect cells, Sf9, directly solubilized and incorporated into nanodiscs^19^. The method was used to retain the native phospholipid composition of the Sf9 microsomal membrane, as well as the activity of cytochrome P450 as analyzed through substrate binding studies. Furthermore, it was estimated to obtain one cytochrome P450 protein embedded nanodisc every 10 nanodiscs. Additionally, Shirzad-Wasei *et al*. reported similar methods for the protein rhodopsin from bovine rod outer segment membranes^17^. On the other hand, nanodisc-like lipid particles stabilized by the styrene maleic acid (SMA) copolymer have been shown to extract membrane proteins from artificial^20,21^ and biological membranes^22,23^, via electrostatic interactions and hydrophobic effects^24^. However, the conditions in which the SMA co-polymer-protein-lipid complex (SMALPs) can be studied are strongly limited by the solubility of the SMA copolymer, which is influenced by the pH of the surrounding environment. Moreoever, while the size and shape of SMALPs have been reported to be reasonably consistent, it is unclear how many SMA polymers surround a single particle. On top of that, the influences of the SMA polymer chain length and the molecular weight distribution of SMALPs remains unclear.

In this study, the aim was to produce nanodisc complexes that can extract the seven-transmembrane helix (7TM) integral membrane protein bacteriorhodopsin (bR) from its native purple membrane (PM) while maintaining the native protein conformation and oligomeric state. Due to the existing methods not being able to efficiently assemble functional trimeric bR embedded nanodiscs directly from the purple membrane, in this study a covalently circularized nanodisc (cND) based method is described. This method was used to extract and incorporate the bR trimer from the PM directly into uniform lipid nanodiscs, under high-salt conditions, with minimal exposure to detergent, without the additionally requirement of synthetic lipids or extra lipid extracts. Using spectroscopic characterization, the trimeric structure and function of bR were observed, in addition to the preservation of the essential PM lipids in the nanodisc. This approach was able to successfully transfer up to 38% of trimeric bR from PM into nanodiscs after optimization and purification.

Natively bR forms a trimer on the PM, and together with ten lipids per bR it forms a hexagonal crystalline lattice across the membrane^25,26^. During the process of proton pumping, bR undergoes a photocycle that consists of different intermediate states with varying absorption wavelengths due to conformation changes and protonation/deprotonation of the retinal Schiff base. In particular, the transition to the M state is known to be related to the deprotonation of the Schiff base and the subsequently release of the proton to the extracellular side, while the O state indicates the relaxation of the retinal before the next photocycle begins^27,28^. bR has previously been reported to incorporate into lipid nanodiscs; however, the studies were done using synthetic lipids and including solubilizing bR with detergent before nanodisc assembly^5,8,9,29,30^. By observing these photocycle intermediates initiated by a pulsed laser, we showed that the function and activity of membrane protein embedded in the nanodisc, assembled directly from the native membrane, were able to be maintained.

## Experimental Procedures

### Preparation of membrane scaffold proteins

Membrane scaffold proteins MSP1E3D1, and MSP1E3D1_srt, which contains an additional his tag and a sortase recognition site, were expressed using Rosetta 2(DE3) with a pET28 vector. The bacteria were grown using either a 2 L or 6 L Winpac Bench-Top fermenter (Major Science, Taiwan) at 37 °C in LB medium. The cultures were grown to an optical density (OD_600_) of 0.8–1.0 before inducing with 1 mM isopropyl β-D-1-thiogalactopyranoside. The bacterial cells were harvested 4–5 h after induction, and the bacterial pellet was kept at −80 °C until purification. The purification procedure was carried out as previously described^31^. The sortase catalyzed circularization reaction to produced cE3D1 has also been previously described^32^, with modifications described by Yusuf *et al*. utilizing a detergent assisted method^33^. MSP1E3D1_srt contains a his-tag on both the N- and C- terminus, with an additional sortase recognition site, LPGTG, located in the N-terminus. The N-terminal his-tag was cleaved by the TEV protease to expose the sortase recognition site. The cleaved MSP1E3D1_srt(−) was then diluted in 50 mM Tris-HCl, 150 mM NaCl, and 2 mM Triton X-100 at pH 7.5. The mixture was then added dropwise into the sortase reaction mixture containing 3 μM of evolved sortase A (SrtA), 50 mM Tris-HCl, 150 mM NaCl, 1 mM Triton X-100, and 10 mM CaCl_2_. The final ratio between MSP1E3D1_srt and SrtA was 1 to 1. The reaction was stirred at room temperature for 16 h before the sample was purified using Nickel nitrilotriacetic acid (Ni-NTA) beads to remove SrtA, TEV protease, and any unreacted MSP1E3D1_srt(−). The Ni-NTA flow through was collected, concentrated, and the detergent removed by an overnight treatment with BioBeads (Bio-Rad Laboratories, USA).

### Preparation of PM

*Halobacterium salinarum* S9 was grown as previously described, with no additional antibiotics necessary due to the culture conditions being too harsh for any contamination^34^. The expression culture was grown at 38 °C with illumination in either an Erlenmeyer flask or a Winpact Bench-Top Fermenter (Major Science, Taiwan). The PM was purified from the bacterial pellet using a standard sucrose gradient procedure, in an Optima XE-90 Ultracentrifuge with the SW28 swing bucket rotor (Beckman Coulter, USA) spinning at 87300 × *g* for 22 h (Supplementary Figure S1). After the overnight centrifugation, the PM was extracted and underwent a fast centrifugation in an Avanti J-26S XP Centrifuge with JA25.50 rotor (Beckman Coulter, USA) at 27216 × *g* for 1 h to remove any residual sucrose. The PM pellet was then resuspended in water and gently sonicated using a Q700 sonicator (QSonica, USA). The concentration of PM was subsequently estimated with steady-state spectroscopy, using the absorption at 568 nm and the extinction coefficient of bR ε_568_ = 62700 M^−1^ cm^−1^ ^35^. The PM was then stored at 4 °C.

### PMND assembly

The assembly of native nanodiscs was based on a protocol previously described^19^, with additional modifications and optimization. The native purple membrane nanodisc (PMND) does not require the target protein to be previously isolated and solubilized, nor does it require additional synthetic lipids or lipid extracts. The concentration of bR in PM for the PMND assembly was maintained at 25 μM in all assembly conditions. The detergent, Triton X-100, was added separately to mildly interrupt the inter-trimeric configuration. The assembly buffer contained 25 mM Tris-HCl, 0.5 mM EDTA at pH 7.5, and NaCl ranging between 100 mM to 5 M depending on the optimized conditions. The different pH conditions were achieved by adjusting the Tris based buffer to pH 9, 7.5, and 6, furthermore a sodium acetate-based buffer was used for assembly at pH 5. Additional details of the assembly are found in the supplementary information. After assembly the sample was then placed at 4 °C with gentle agitation, before BioBeads were added to remove the detergent. The BioBeads were separated from the assembly using centrifugation at 3381 × *g*. Any additional precipitate in the solution, such as free PM, was removed by centrifugation at 21130 × *g* for 10 mins. The PMND sample was then purified using size exclusion chromatography.

### Size exclusion chromatography

The PMND sample was purified using size exclusion chromatography, performed with either a HiLoad 16/600 Superdex 200 PG column or a Superdex 200 Increase 10/300 GL column (GE Healthcare, USA) depending on the volume and concentration of the sample for optimal separation. Experiments were performed at room temperature. The elution buffer consisted of 25 mM Tris, 100 mM NaCl, and 0.5 mM EDTA at pH 7.5. Sample elution was monitored by the protein absorption peak at 280 nm and the characteristic absorption of the retinal state of bR at 560 nm.

### Steady state absorption spectroscopy

The PMND sample was light adapted for more than 45 min prior to the steady state absorption spectroscopy. The steady-state UV-Vis absorption spectra were recorded with a USB4000-UV-VIS spectrometer (Ocean Optics) at room temperature. The path length of the cuvette used was 1 cm.

### Circular dichroism

The circular dichroism spectra were recorded with a J-815 spectrometer (JASCO, Japan), averaged for 1.2 s, at 1 nm intervals between 700 nm to 400 nm at 20 °C. The path length of the cuvette used was 1 mm.

### Zernike phase transmission electron microscopy (TEM)

The samples were placed on grids and air dried without staining. Phase TEM images were taken in an in-focus condition at 200 kV using a JEM2100 TEM (JEOL, Japan) equipped with a LaB6 filament, using a Zernike phase plate with a ~700 nm central hole, and a direct detector DE-12 (Direct Electron, USA). Images were recorded at nominal microscope magnifications of ×100 and ×200.

### Transient absorption spectroscopy

The PMND sample was light adapted for more than 30 min prior to the transient absorption experiment. The concentration of PMND was maintained at 15 μM by observing the maxima absorbance in the steady state absorption experiment. Fractions 1 to 6 from the size exclusion profile, which consisted of only trimeric bR, were collected, concentrated and exchanged into a buffer containing 0.5× phosphate buffered saline (PBS), with an additional 100 mM NaCl at pH 5.8. The pulsed laser excitation at 532 nm was generated by the second harmonic neodymium-doped yttrium aluminum garnet (Nd:YAG) laser system (LS-2134UTF, Lotis Tii, Belarus). The laser flux was monitored at 0.37 mJ cm^−2^ in front of the sample compartment, while the laser repetition frequency was optimized to avoid overshooting (supplementary Figure S2). The final repetition frequency was 0.5 Hz, and each spectrum was averaged over 500 excitations. The probing light was setup perpendicular to the excitation laser beam and was provided by a deuterium tungsten halogen lamp DH2000 (Ocean Optics), combined with a neutral density filter (Newport, USA) as well as other color filters. After passing through the sample compartment, the probe beam was further dispersed by a monochromator (McPherson, USA) for detection of the parent depletion (560 nm) and the intermediates M (410 nm) and O (670 nm). The path length of the cuvette was 1 cm and all experiments were performed at 20 °C. The optical modulation was monitored using a R928 photomultiplier (Hamamatsu, Japan) and recorded with an oscilloscope WaveSurfer 2MXs-B (LeCory, USA). The temporal profiles were averaged over 300 laser excitations to increase the signal-to-noise ratio. The change of the absorbance (ΔAbs.) was calculated using the following equation:

$${\rm{\Delta }}\mathrm{Abs}.=\,-\,\mathrm{log}({{\rm{S}}}_{{\rm{t}}}/{{\rm{S}}}_{0})$$

where S_t_ and S_0_ represent the DC coupled voltages in the presence and the absence of the excitation laser, respectively.

### Lipid extraction

Lipids from the PM and the PMND were extracted using an organic solvent method. The PM (400 μL) was mixed with chloroform and methanol at a 0.8:2:1 ratio. The sample was then vigorously shaken, sonicated, and centrifuged to achieve clear phase separation. The upper water phase was discarded, before 500 μL of chloroform and water were added to the lower organic solvent phase, to obtain a two-phase system. The sample was then placed in the dark at room temperature. The chloroform phase was then dried using N_2_ gas.

### ^31^*P* NMR spectroscopy

The ^31^P NMR sample was prepared using a methanol reagent containing D_2_O and dissolved Cs-EDTA. Caesium salt EDTA was used due to the poor solubility of the sodium salt EDTA in organic solvents, and was prepared by titrating EDTA free acid into distilled water with solid CsOH until pH 6 was reached. The solution was then dried by lyophilization and dissolved in D_2_O to a concentration of 0.2 M. The methanol reagent was prepared by adding D_2_O-Cs-EDTA to methanol in the ratio of 1 to 4. For the ^31^P NMR sample, dried extract lipids were dissolved in 400 μL of deuterated chloroform and mixed with 200 μL of the methanol reagent containing Cs-EDTA. The sample was then placed in the NMR tube where two phases could be observed, a major chloroform phase and a smaller water phase. The ^31^P NMR spectra were recorded using a Bruker Avance III 500 MHz NMR spectrometer (Bruker, USA) equipped with a 5-mm CryoProbe Prodigy probehead. The experiment was performed at the Instrumentation Centre of National Taiwan University, Taipei, Taiwan.

### Liquid chromatography electron spray ionization mass spectrometry (LC-ESI-MS)

Dried lipid extracts were dissolved in methanol. Samples were detected by LC-ESI-MS on an LTQ Orbitrap XL ETD mass spectrometer (Thermo Fisher Scientific, USA) equipped with Waters Acquity UPLC (Waters, USA), using a ZIC-HILIC column (1.0 mm × 150 mm, 3.5μm, Merck, Germany). Briefly, a gradient from 98% buffer B at 2 min to 60% buffer B at 45 min with a flow rate of 50 μL/min was employed, where buffer A was 0.1% formic acid/H_2_0 and buffer B was 0.1% formic acid/acetonitrile. The MS conditions were a mass range m/z 320–2000 and a resolution of 30,000 at m/z 400. The electrospray voltage was maintained at 4.0 kV and capillary temperature was set at 275 °C. The experiment was performed at the Genomic Research Centre at Academia Sinica, Taipei, Taiwan.

## Results and Discussion

### Assembly of native purple membrane nanodiscs

**Figure 1:**
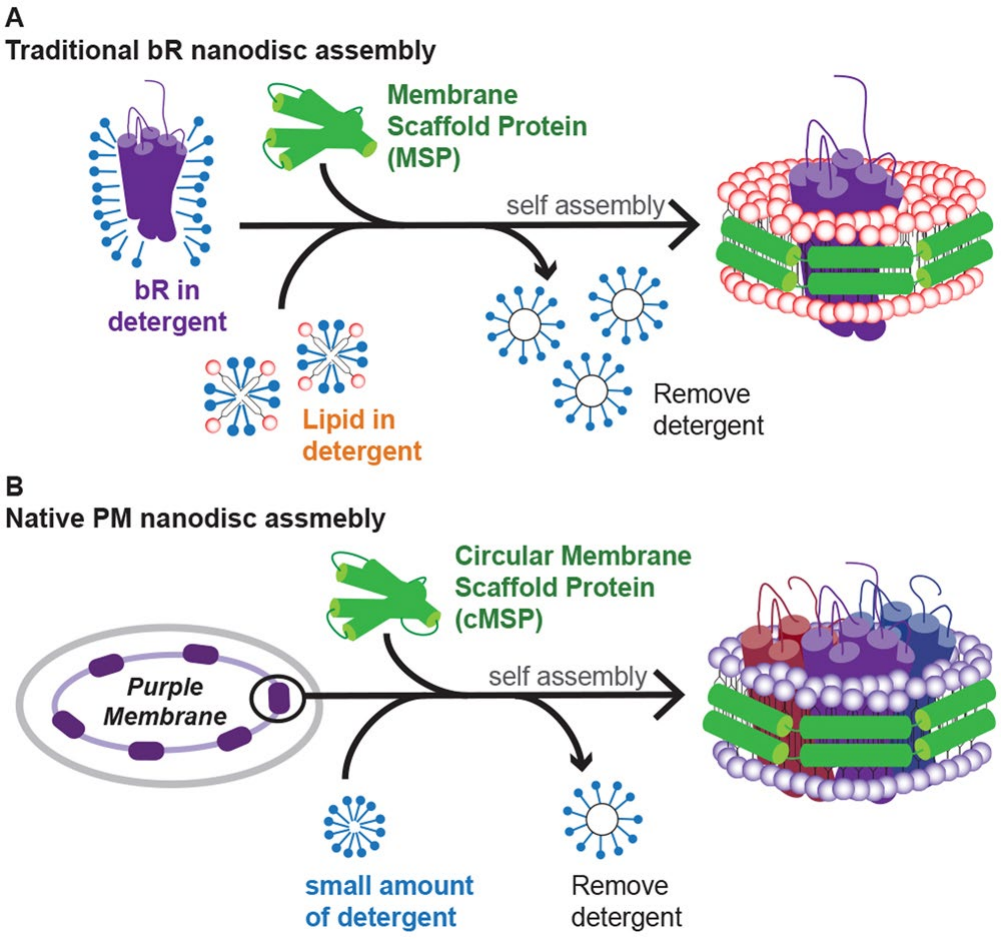
Schematic diagram of the nanodisc assembly producing (**A**) bR embedded nanodiscs and (**B**) native PM nanodisc assembly. The native PM nanodisc was assembled using a circular MSP with covalently joined N- and C-termini, without any synthetic lipids or additional lipid extracts. A small amount of detergent was added into the system to mildly disrupt the membrane and was removed from the system after a short period of time using BioBeads. Individual bRs were colored differently to emphasize the trimeric conformation.

**Figure 2:**
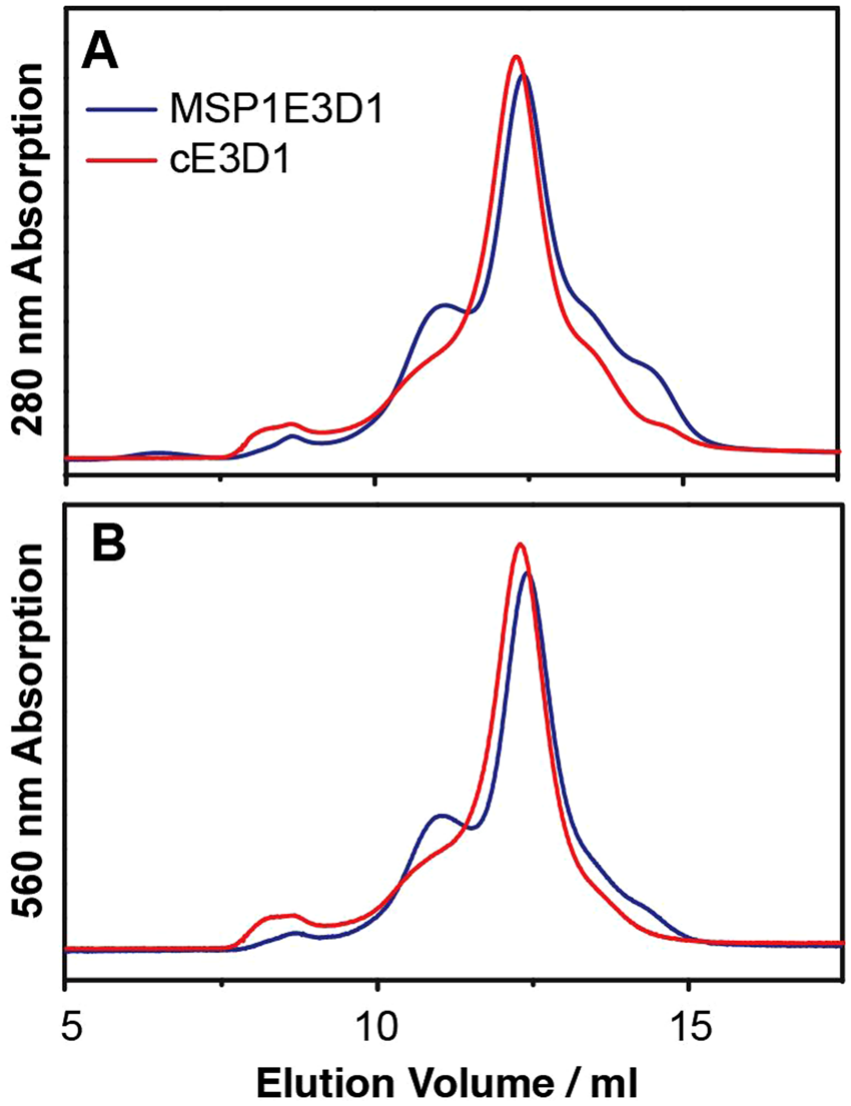
Size exclusion chromatography profiles of PMND assembled using convention linear MSP1E3D1 (blue) and circularized cE3D1 (red). These were monitored at (**A**) 280 nm and (**B**) 560 nm absorption. Both size exclusion profiles were recorded with the same volume of sample injected, and both samples were previously optimized and assembled using the same PM and NaCl concentration.

The assembly of the native membrane nanodisc, as first reported by Civjan *et al*.^19^, differs from the traditional nanodisc assembly as seen in Fig. 1. In traditional nanodisc assembly (Fig. 1A), the membrane protein of interest is usually isolated and often in detergent micelle, and the extrinsic lipid molecules are added to the assembly mixture before the removal of detergent to initiate nanodisc self-assembly. However, in this study the assembly of native PMND was performed without prior solubilization of the target membrane protein (Fig. 1B). A small amount of detergent, Triton X-100, was added to mildly perturb the inter-trimeric interaction and was removed from the system using BioBeads SM-2 adsorbents (Biorad Laboratories Inc., CA) after a short period of time, as brief as 10 min, to prevent disruption to the intra-trimeric configuration. Long-term exposure of membrane proteins to detergents should be avoided, for example G-protein couple receptors have been reported to have a time-dependent loss of activity in detergent^36^. In this study, circular membrane proteins with covalently joined N- and C- termini from the sortase A catalyzed reaction were used as the membrane scaffold protein^32,33^. It was demonstrated that circular MSP1E3D1 (cE3D1) could extract bR from PM to form PMND more efficiently than the linear version without undesirable bR aggregation (Fig. 2). Additionally, compared to the elution profile of the nanodisc made with the linear MSP1E3D1, the peak of the nanodisc made with cE3D1 was more symmetrical and had less undesirable aggregations. Furthermore, the native membrane nanodisc was assembled without the addition of any synthetic or previously extracted lipid molecules. Size exclusion chromatography was applied to remove the undesirable aggregation, particularly those appearing at a higher molecular weight, that may arise from small patches of purple membrane and other lipid-protein or protein-protein aggregation. The aggregation smaller than PMND may be due to excess membrane scaffold proteins.

**Figure 3:**
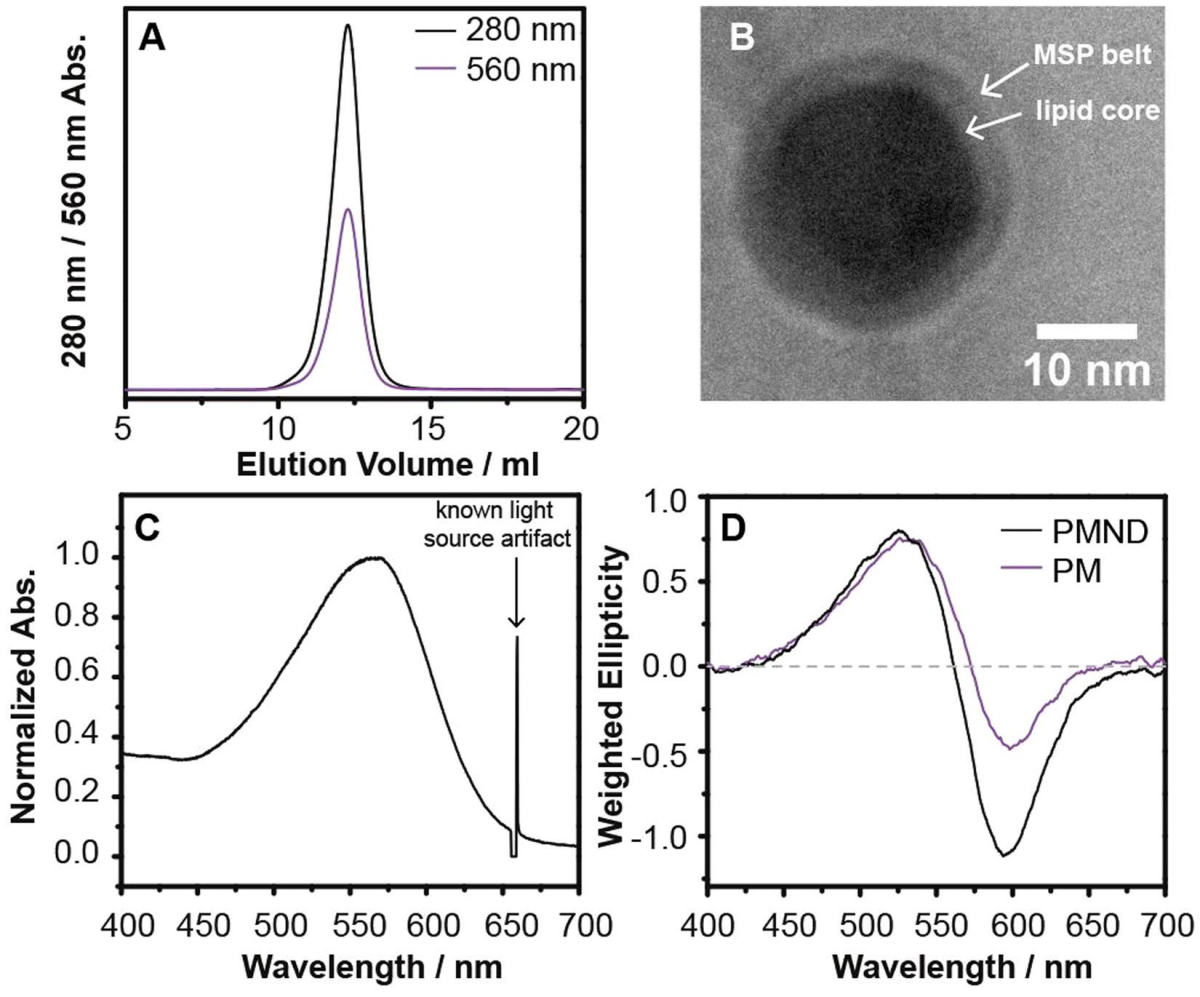
Characterization of the optimized and purified PMND. The characterization was performed using (**A**) size exclusion chromatography, (**B**) high resolution Zernike phase TEM, (**C**) steady-state absorption spectroscopy and (**D**) CD in the visible wavelength range and compared to that obtained from the PM.

The optimization and the subsequent formation of the native PMND were analyzed using size-exclusion chromatography (SEC) with dual wavelength detection at both 280 nm and 560 nm, the latter being the characteristic absorption of functional bR. After the optimization of the nanodisc assembly conditions (supplementary Figure S3), the elution profile of the optimized and purified PMND showed sharp and symmetrical peaks detected at both 280 nm and 560 nm (Fig. 3A), indicating that the products were similar in size. The SEC fractions that contained trimeric bR in the PMND were combined and were subjected to sodium dodecyl sulfate polyacrylamide gel electrophoresis (SDS-PAGE) to verify the presence of both bR and the membrane scaffold protein cE3D1 (supplementary Figure S4). Using the various extinction coefficients of PM at 280 nm^37^ and 560 nm^35^, and cE3D1 at 280 nm, and the ratio of absorption at 560 nm to 280 nm of PMND, it was calculated that approximately 47% of nanodiscs were embedded with bR in this study. Furthermore, high resolution Zernike phase TEM^38,39^ images of unstained PMND showed perfect disc-shaped units, with a clear differentiation between the MSP belt and the core lipid components (Figs 3B and S5). It is worth noting that negative staining commonly used on TEM specimens was not required while preparing our PMND TEM specimens, as the high-resolution TEM images shown in this work were acquired by using a Zernike phase plate to improve contrast. Therefore, any possible imaging artifacts or disturbance caused by the staining procedure was avoided. Overall, these results indicate successful nanodisc formation containing bR assembled directly from native PM. On the TEM images, the nanodisc sizes are shown to be greater than 20 nm in diameter. The reported sizes of nanodisc composed of linear MSP1E3D1 are 13 nm^40^. PMND appeared to be assembled in a larger nanodisc, possibly to accommodate the trimeric structure of bR. Interestingly, larger discs have been unstable compared to the smaller discs, and yet PMND showed remarkable stability, suggesting perhaps trimeric bR might stabilize the larger nanodisc structure.

The steady-state absorption spectrum of PMND showed a characteristic band at 563 nm after light adaptation. When compared to the absorption spectrum of solubilized bR in Triton X-100 at 553 nm^41^ and the monomeric bR in synthetic lipid nanodiscs at 560 ± 1 nm^8^, PMND displayed a much smaller spectral shift from the native trimeric bR in PM at 568 nm (Figs 3C and S6)^41,42^. This suggests that the tertiary structure and lipid environment of bR in PMND is even closer to bR in PM compared to bR in synthetic lipid nanodiscs.

Circular dichroism (CD) spectroscopy at the wavelength of visible light was used to identify the oligomeric state of bR. CD was used as the trimeric bR in PM would show a biphasic feature with a positive band around 530 nm and a negative band at around 600 nm, whereas the monomeric bR would only show a monophasic CD signal at the visible light wavelength^43,44^. The weighted CD spectrum of PMND (Fig. 3D, black) displayed a clear biphasic feature, showing that the trimeric conformation adopted by bR in native PM was preserved after the transfer to PMND. When compared to the CD spectrum recorded for PM (Fig. 3D, purple), it is seen that while the biphasic features characteristic of the trimeric conformation are observed, the ratio between the positive and the negative peaks are different between the PM and PMND. In particular, the amplitude of the positive peak remained similar, however the amplitude of the negative peak of PMND significantly increased. This difference may be attributed to the difference in inter-trimeric interactions in PM and PMND, with bR existing as an isolated trimer in the PMND it would not have an inter-trimer influence on the CD spectrum^45^.

**Figure 4:**
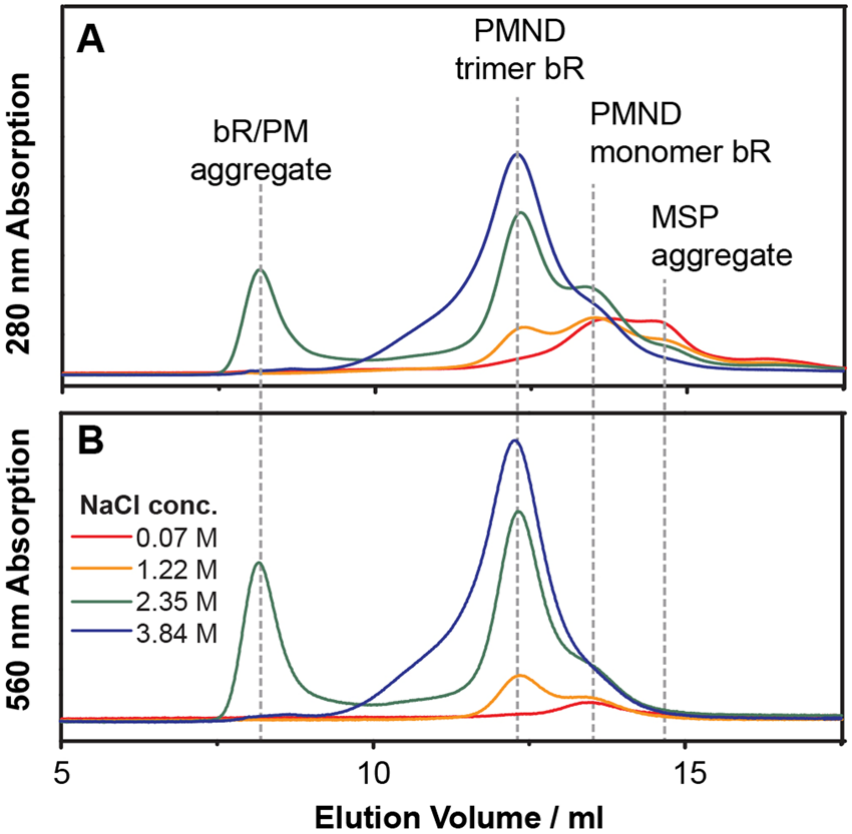
Size exclusion chromatography profiles of PMND assembled with different NaCl concentrations. Sample elution was monitored at (**A**) 280 nm and (**B**) 560 nm. Peak assignment was performed using the 560 nm absorption as well as estimated molecular weight from the elution profiles.

**Figure 5:**
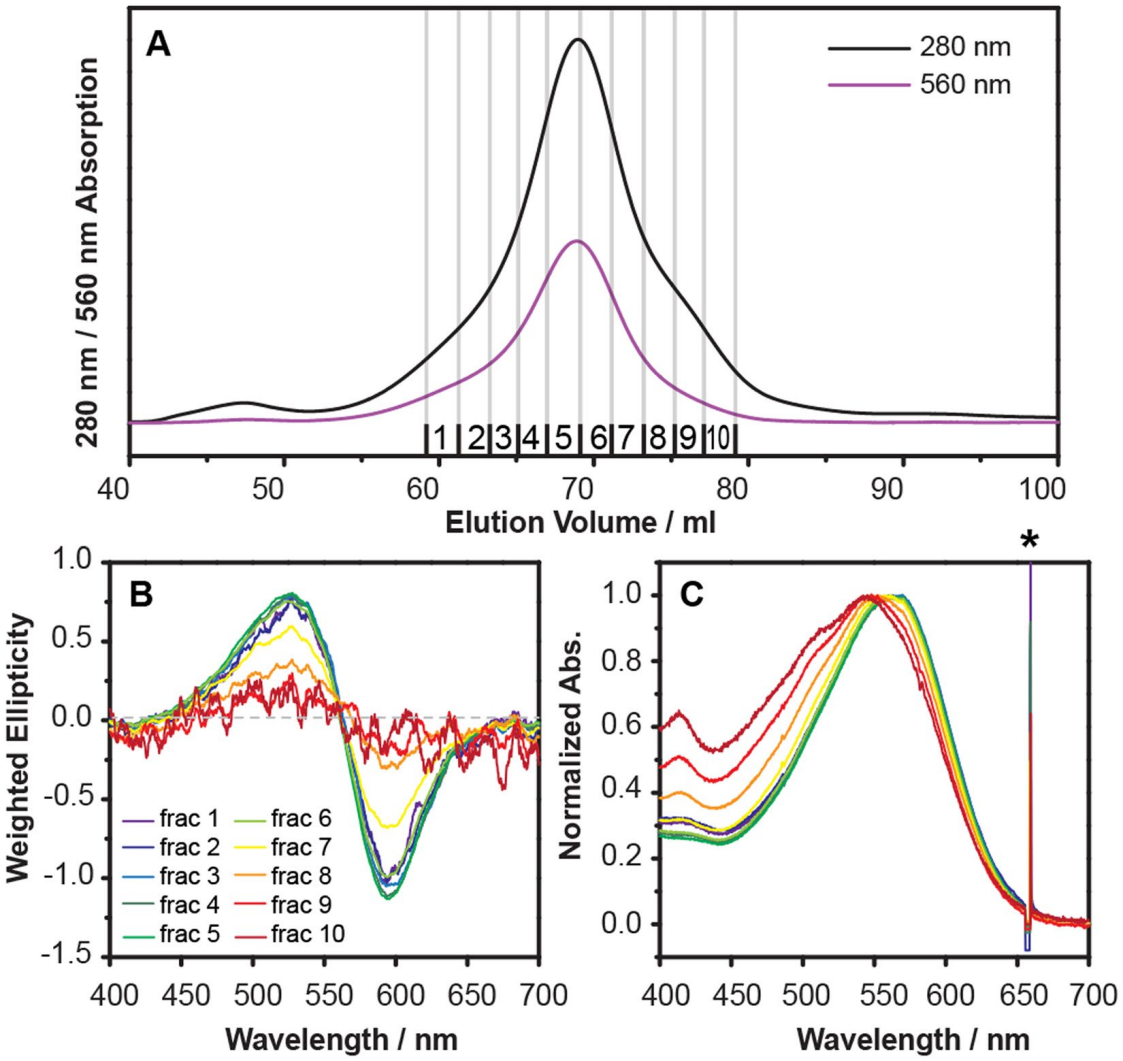
Separation of trimeric and monomeric bR in PMND using size exclusion chromatography. (**A**) The sample was first purified using size exclusion chromatography and the fractions collected were concentrated individually. (**B**) These fractions were then characterized by CD spectroscopy in the visible wavelength range. Each circular dichroism spectrum was weighted by its respective bR concentration, which was determined using absorbance of steady-state absorption spectroscopy. (**C**) The fractions were also characterized by steady-state absorption spectroscopy. Normalized steady state absorption spectra showed shifts in absorption maxima, where * indicates a known artifact of light source from experimental setup.

Previous studies have shown that bR can form either a monomer or a trimer when reconstituted into lipid vesicles or 1,2-dimyristoyl-sn-glycero-3-phosphocholine nanodiscs at different temperatures^46,47^ or with different bR to lipid ratios^5,48,49^. It has also been demonstrated that oligomeric states of bR in membrane bilayer mimics can be altered by adjusting the NaCl concentration^50^ and with the addition of PM polar lipids^20,51^. As the extraction and incorporation of bR and the PM native lipids into the nanodiscs was done in a single step, there was no need to adjust the type or amount of lipid incorporated. However, the final concentration of NaCl needed to be adjusted during the PMND assembly prior to the SEC purification (Fig. 4). In this study it was found changes to the NaCl concentration not only affected the oligomeric state of bR in PMND, but also significantly influence the yield of PMND. By increasing the NaCl concentration present in the PMND assembly to 3.84 M, a production yield of up to 38% of trimeric bR in PMND from PM was able to be obtained. This was estimated using the bR characteristic absorption, and the calculation is further detailed in the supplementary data. This reported yield consists solely of trimeric bR in PMND, due to PMND assembly yielding nanodiscs that contain mainly trimeric bR with only a minor population of bR in monomeric form. Though in this study, the two species could be successfully separated using SEC (Fig. 5). The visible CD spectra of the fractions collected from the SEC profile (Fig. 5A), were weighted using the bR concentration of the respective fraction. The CD spectra (Fig. 5B) showed consistent biphasic signals with similar amplitudes and intensities in the earlier fractions. However, the biphasic feature rapidly decreased in fraction 7 and was then unable to be observed in fraction 9. This indicates that the assembled PMND collected in fractions 1 to 6 contained consistent trimeric bR populations, while fraction 7 to fraction 10 contained less trimeric bR species. However, the bR characteristic absorption was still observed, and therefore suggests the existence of functional bR but in a non-trimeric form. This finding was further supported by the steady-state absorption spectra (Fig. 5C), where the absorption of PMND in fractions 1 to 6 were identical, however the absorption maxima began to exhibit a blue shift towards 550 nm from fraction 7, previously reported to be the absorption of monomeric bR^41^. These results suggest that the PMND obtained in the early fractions were consistent and homogenous, while the later fractions contained a mixed population of bR oligomeric states. Therefore, only PMND samples obtained in the early fractions collected during the SEC elution were subjected to further characterization. Additionally, size exclusion profiles of PMND assembled with different pHs, from 5 to 9 showed that the yield of PMND production was not significantly influenced by the pH during the assembly, highlighting that pH was not a limiting factor to the success of PMND assembly (supplementary Figure S7).

These results show that direct assembly using a cND based method can incorporate bR into a nanodisc without the requirement of liposome reconstitution or detergent solubilization. Moreoever, they show that homogenous trimeric bR conformation is possible, where previous methods of bR assembly in MSP based conventional nanodisc or SMALPs reported either having a mixed population^5^ or primarily monomeric species present^8,9,20,21^. In this study, after optimization and purification, the production of PMND yielded a sharp symmetrical peak of a homogenous population of isolated trimeric bR in nanodiscs. Furthermore, high resolution TEM images of unstained samples showed consistent disk-like features with similar sizes.

### Lipid content and protein function

**Figure 6:**
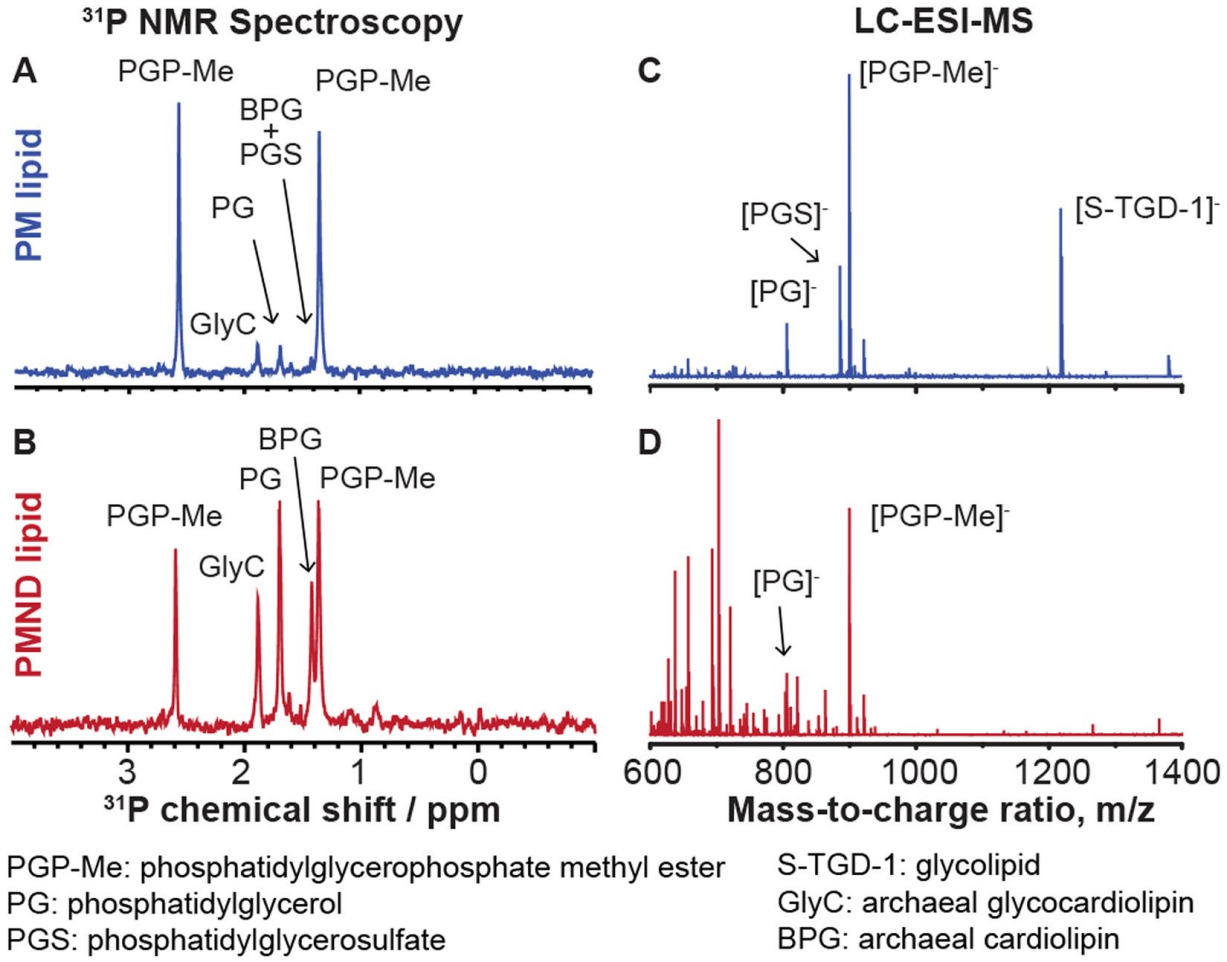
Lipid analysis of PM and PMND lipid extracts. (**A–B**) The lipid extracts were characterized using^31^P NMR spectroscopy. (**C–D**) In addition they were characterized by LC-ESI mass spectrometry. Assignment of ^31^P chemical shift and molecular weights were performed as previously described by Corcelli *et al*.^26^.

The lipid content of the PM was shown to be unique, and capable of influencing the photocycle activity of bR. In particular, phosphatidylglycerophosphate methyl ester (PGP-Me) has previously been suggested to be essential for the conformation and photocycle of bR and the stability of PM^52–54^. Since the majority of the lipids found in the PM have been phospholipids^26,55^, in this study ^31^P nuclear magnetic resonance spectroscopy (NMR) was combined with liquid chromatography-electrospray ionization-mass spectrometry (LC-ESI-MS) for the lipid content analysis (Fig. 6). In the ^31^P NMR spectra two lipid types were observed, archaeal cardiolipin and archaeal glycocardiolipin, which was not detected in the mass spectra. Any additional LC-ESI-MS signals observed were attributed to protein fragmentation during the lipid extraction procedure. The ^31^P chemical shifts and lipid molecular weights were assigned according to previous research^26^. Therefore, the lipid analysis results suggest that the essential PM lipids, PGP-Me, and most other phospholipids, were successfully transferred from PM to the PMND. Compared to the other phospholipids, PGP-Me may have a better transfer efficiency from PM to PMND due to its position within the trimeric bR^56^. Since the trimeric conformation of bR on PM is maintained, the intratrimeric lipid molecules may be maintained as well.

**Figure 7:**
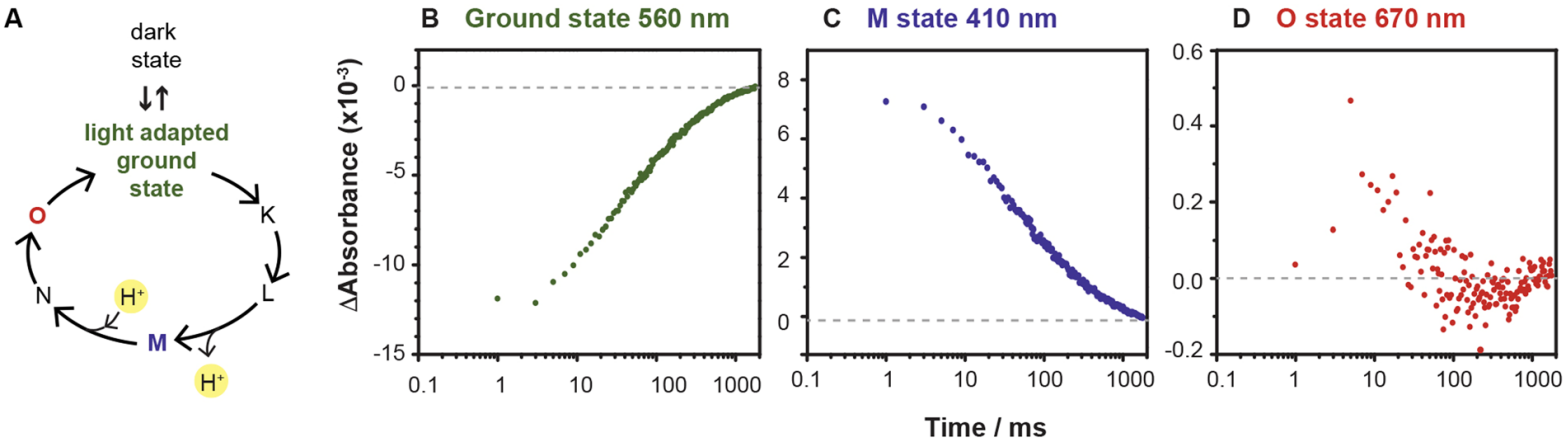
(**A**) The main intermediate states of the bR photocycle, with the intermediate states observed in this work highlighted in color. (**B–D**) Temporal profiles of (**B)** ground state recovery at 560 nm, (**C**) M state intermediate at 410 nm and (**D**) O state intermediate at 670 nm upon photoexcitation of the PMND.

The function of bR was measured using the activity of the photocycle initiated by a pulsed illumination at 532 nm and determined using time-resolved difference absorption spectroscopy. The main intermediate states of the bR photocycle (Fig. 7A) have been previously described^27,28^. The recovery rate of trimeric bR in PMND was monitored at 560 nm, the kinetics of the proton-pumping M intermediate were measured at 410 nm, and the formation and population of O intermediate were observed at 670 nm. The time-resolved spectra of PMND at the previously mentioned wavelengths upon laser excitation, manifested full recovery of the bR ground state, as well as a population of the key intermediate states M and O (Fig. 7B–D). These results indicate that the function and proton pumping activity of bR were preserved, with bR in PMND undergoing a photocycle with a pathway similar to that observed in PM.

## Conclusions

In this study, an efficient assembly of PMND, in which up to 38% of 7TM bR trimer was directly extracted and assembled into covalently cND from the purple membrane, was achieved. In addition, PMND was demonstrated to assemble under a wide range of pHs, with well-preserved lipid contents and activity of the trimeric bR. Furthermore, the high production yield of a homogenous sample achieved in this study is ideal for additional biophysical studies, to further improve the quality of the sample and experimental results. This highly efficient approach provides flexibility over preparation conditions and potentially could be used as an alternative platform for membrane protein characterization.

## Electronic supplementary material

Supplementary Information
