# Supplementary material for: Highly Efficient Transfer of 7TM Membrane Protein from Native Membrane to Covalently Circularized Nanodisc
Source: Sci Rep. 2018 Sep 10;8:13501. doi: 10.1038/s41598-018-31925-1 (PMC6131177; doi:10.1038/s41598-018-31925-1)
Supplement: Supplementary file 1 — Supplementary Information [file 41598_2018_31925_MOESM1_ESM.docx]

**Supporting Information**

**Highly Efficient Transfer of 7TM Membrane Protein from Native Membrane to Covalently Circularized Nanodisc**

Vivien Yeh, ^⊥†^ Tsung-Yen Lee,^‡^ Chung-Wen Chen, ^†^ Pai-Chia Kuo, ^§^ Jessie Shiue, ^§^

Li-Kang Chu,*^‡^ and Tsyr-Yan Yu*^†^

^†^Institute of Atomic and Molecular Sciences, Academia Sinica, 1, Sec. 4, Roosevelt Rd., Taipei 10617, Taiwan

^‡^Department of Chemistry, National Tsing Hua University, 101, Sec. 2, Kuang-Fu Rd., Hsinchu 30013, Taiwan

^⊥^Department of Chemistry, National Taiwan University, 1, Sec. 4, Roosevelt Rd., Taipei 10617, Taiwan

^§^Institute of Physics, Academia Sinica, No.128, Sec. 2, Academia Rd., Taipei 11529, Taiwan

* Corresponding authors

**Supporting Experimental Procedure**

*a) PMND assembly*

**Table S1.** PMND assembly and individual components.

|  | Concentration in assembly |
| --- | --- |
| DTT | 1 mM |
| Protease inhibitor | 1× |
| Phosphatase inhibitor | 1× |
| cE3D1 | 20 to 33 μM |
| Triton X-100 | 5 to 20 mM |
| Assembly buffer | Topping up assembly |
| PM | 25 μM |

1. Each component was added into the assembly mixture, except for Triton X-100, thus increasing the preservation of the native conformation. The sample was mixed by pipetting.
2. The detergent was added by diluting a stock of 200 mM Triton X-100 prepared in assembly buffer. The sample was then mixed via pipetting and placed in 4°C in the dark with gentle agitation.
3. BioBeads were then added into the solution to remove the detergent after 10 min of mixing. Approximately 1 mg of Biobeads per 1 mL of PMND assembly mixture was added. The time taken to remove Triton X-100 varied from 4 hours to overnight, to ensure complete removal of the detergent. During detergent incubation and removal, the sample was kept in darkness to prevent light denaturing the bR.
4. The BioBeads were removed as described in manuscript, and the sample was then purified using size exclusion chromatography.

*b) Ratios of bR embedded PMND to empty nanodisc*

**Table S2.** Molar extinction coefficients of PM and cE3D1.

|  | ε_280 nm_ (M^-1^cm^-1^) | ε_560 nm_ (M^-1^cm^-1^) |
| --- | --- | --- |
| PM | 75000^1^ | 62700^2^ |
| cE3D1 | 26930 | - |

A nanodisc consists of two belts of MSP. For an ideal PMND consisting of trimeric bR, the ratio, *R*, of 560 nm absorbance to 280 nm absorbance will be

$$R=\frac{560 nm absorbance}{280 nm absorbance}=\frac{3 \times\varepsilon_{PM, 560 nm}}{3 \times\varepsilon_{PM, 280 nm}+{2 \times\varepsilon}_{cE3D1, 280 nm}}=\frac{188100}{278860}=0.6745$$

An empty nanodisc will not have any absorbance at 560 nm due to the lack of bR. A sample with a mixture of bR embedded nanodiscs and empty nanodiscs will have a lower *R,* as the MSP will still absorb at 280, as seen below:

$$R=\frac{560 nm absorbance}{280 nm absorbanc}$$

$$=\frac{x \left( 3 \times\varepsilon_{PM, 560 nm} \right)}{x \left( 3 \times\varepsilon_{PM, 280 nm}+{2 \times\varepsilon}_{cE3D1, 280 nm} \right)+\left( 1-x \right) \left( {2 \times\varepsilon}_{cE3D1, 280 nm} \right)}$$

where *x* is the fraction of bR embedded nanodiscs with respect to the total nanodisc sample.

The equation can thus be rearranged in order to calculate the amount of bR embedded nanodiscs:

$$x= \frac{{2 R \varepsilon}_{cE3D1, 280 nm}}{\left( 3 \times\varepsilon_{PM, 560 nm} \right)-\left( 3 R \varepsilon_{PM, 280 nm} \right)}$$

*R* of PMND after SEC experiment was determined using steady state absorption spectroscopy to obtain absorbance at 280 nm and at 560 nm, shown in Figure S8.

*c) PMND production yield estimation*

Due to the aggregation nature of bR in PM, absorption at 280 nm can be severely affected by the scattering of light. Therefore, the concentration of bR in PM and PMND were both determined using the absorption at 560 nm. The yield of PMND was calculated by:

$$\frac{mol of bR in PMND collected from SEC}{mol of bR in PM in PMND assembly}$$

It is important to remember that only fractions of PMND consisting of trimeric bR, from size exclusion chromatography experiments, were collected.

**References**

(1) Kalisky, O.; Feitelson, J.; Ottolenghi, M. *Biochemistry* **1981**, *20* (1), 205–209.

(2) Rehorek, M.; Heyn, M. P. *Biochemistry* **1979**, *18* (22), 4977–4983.

**Supplementary Figures**


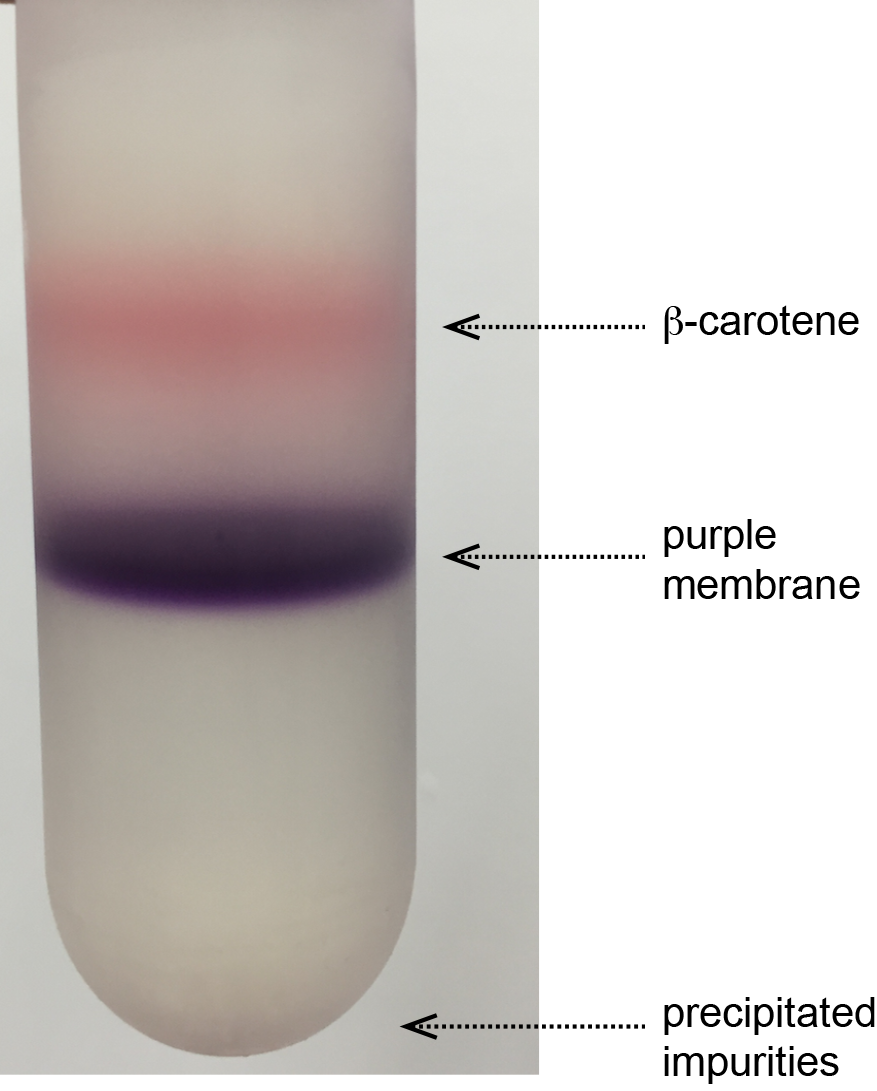


**Figure S1.** Sucrose gradient purification of PM from *H. salinarum*. After overnight centrifugation, only the purple membrane layer was extracted. The sucrose gradient contains distilled water, 30%, 40%, 50%, and 60% sucrose layers.


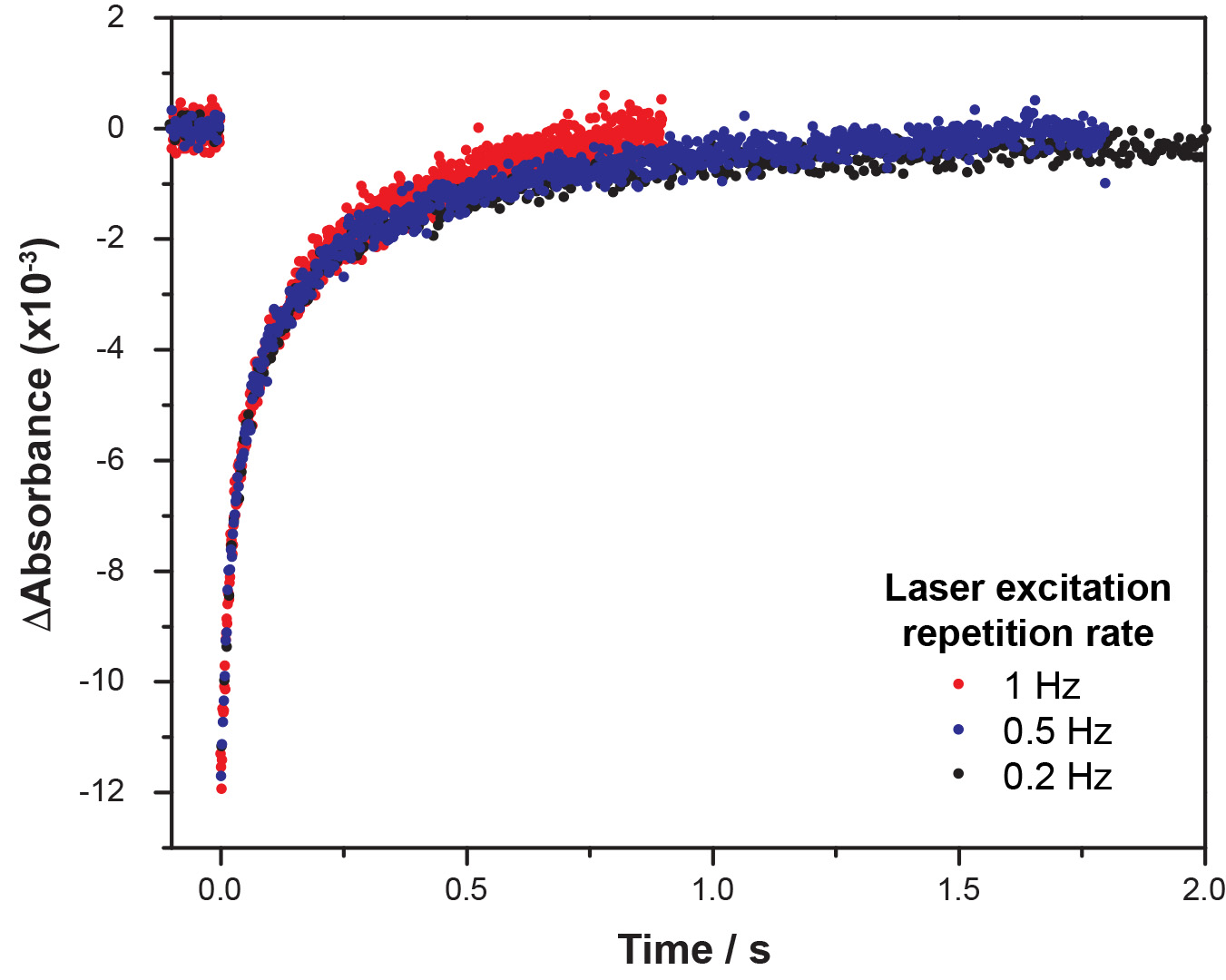


**Figure S2.** Temporal profiles of the recoveries of bR in PMND monitored at 560 nm upon 532 nm pulsed excitation, with repetition rates of 1 Hz (red), 0.5 Hz (blue), and 0.2 Hz (black). The flux of the 532 nm pulsed laser was controlled at 0.37 mJ/cm^2^. The inability to overlap with the slower repetition rate suggests that the photocycle was incomplete after only 1 second, therefore 1 Hz was insufficient to record the complete lifetime of the photocycle.


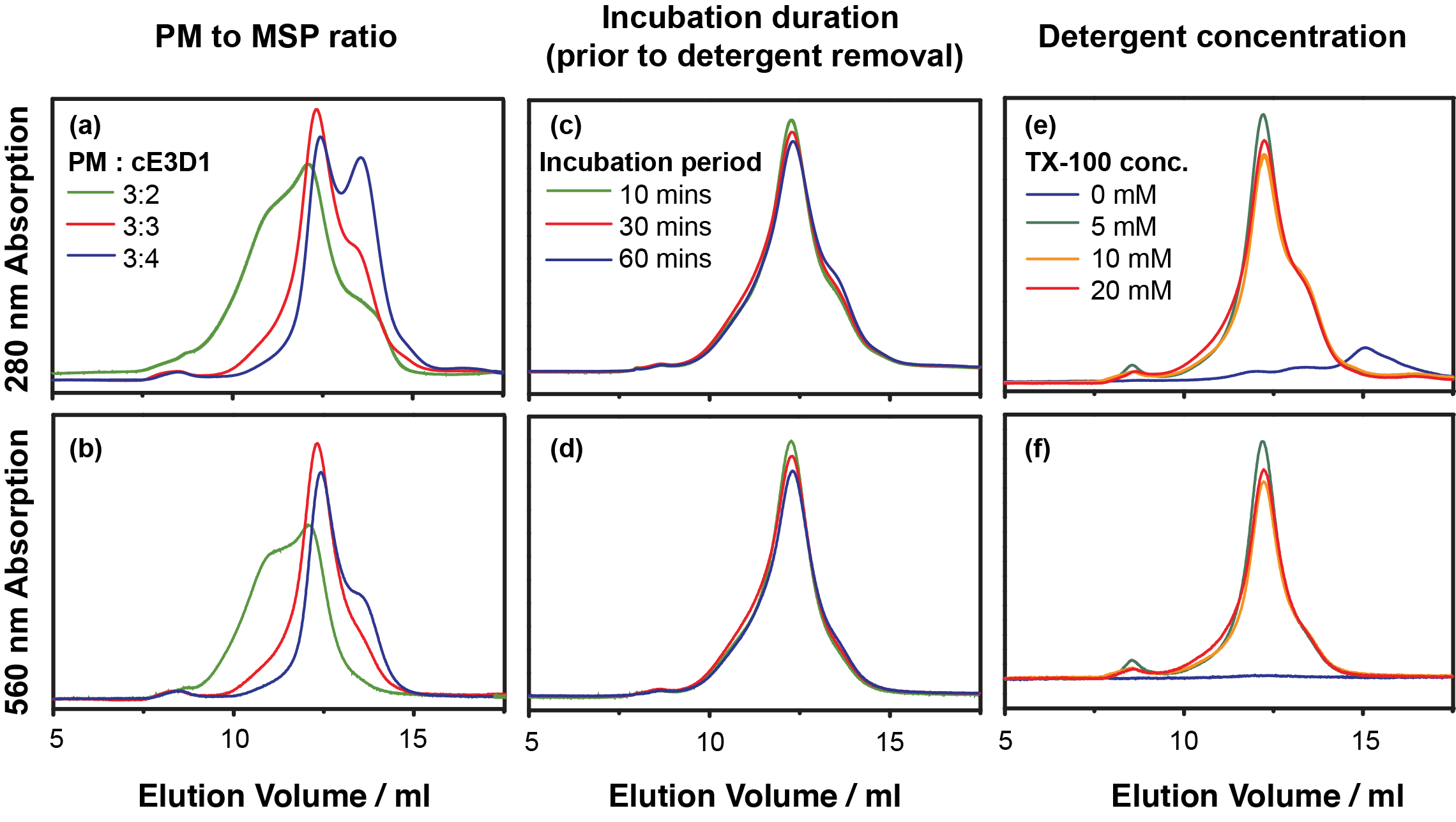


**Figure S3.** Size exclusion chromatography profiles of PMND assembled with (a, b) different PM to MSP ratio, (c, d) different incubation period prior to the removal of detergent, and (e, f) different final concentration of Triton X-100 monitored at (a, c, e) 280 nm and (b, d, f) 560 nm. Each size exclusion profile was recorded by injecting the same volume of sample.


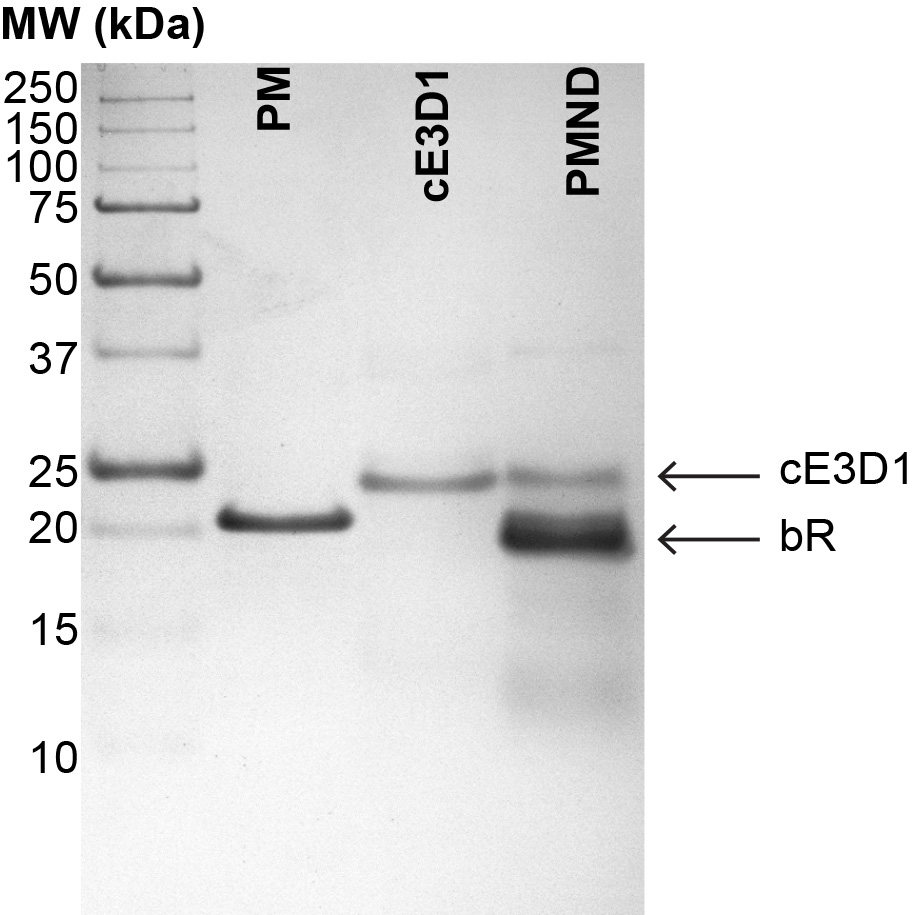


**Figure S4.** SDS-PAGE (5-13%) of PM, cE3D1, and PMND collected from SEC. PMND showed the presences of both cE3D1 and bR, suggesting that the nanodisc was assembled with the target protein, bR, embedded. The uncropped full-length gel image is provided in Figure S4b with the cropped area highlighted in red box.


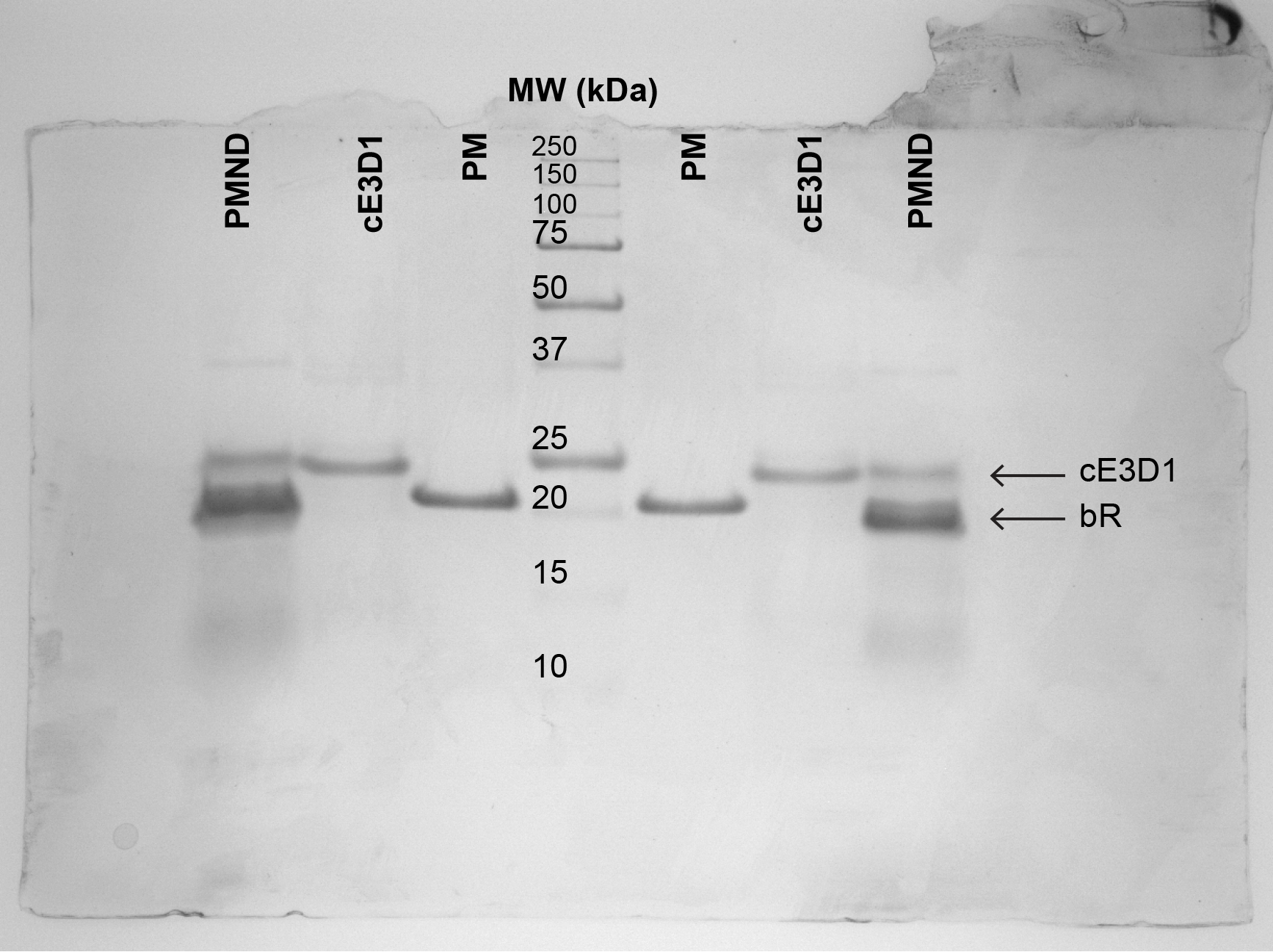


**Figure S4b**. Uncropped full-length SDS-PAGE gel image of Figure S4.


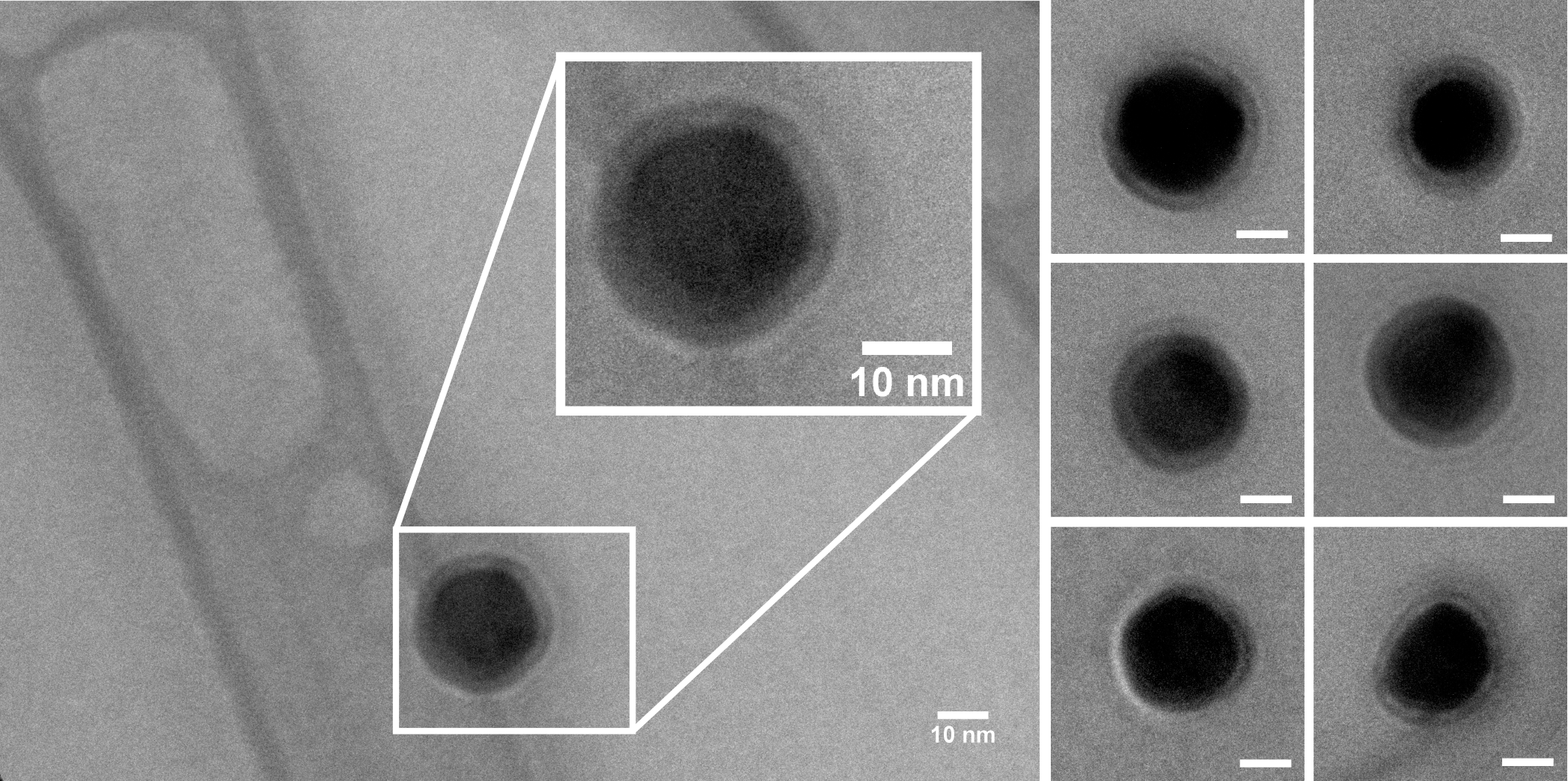


**Figure S5.** Unstained phase TEM images of PMND. Scale bars are 10 nm.


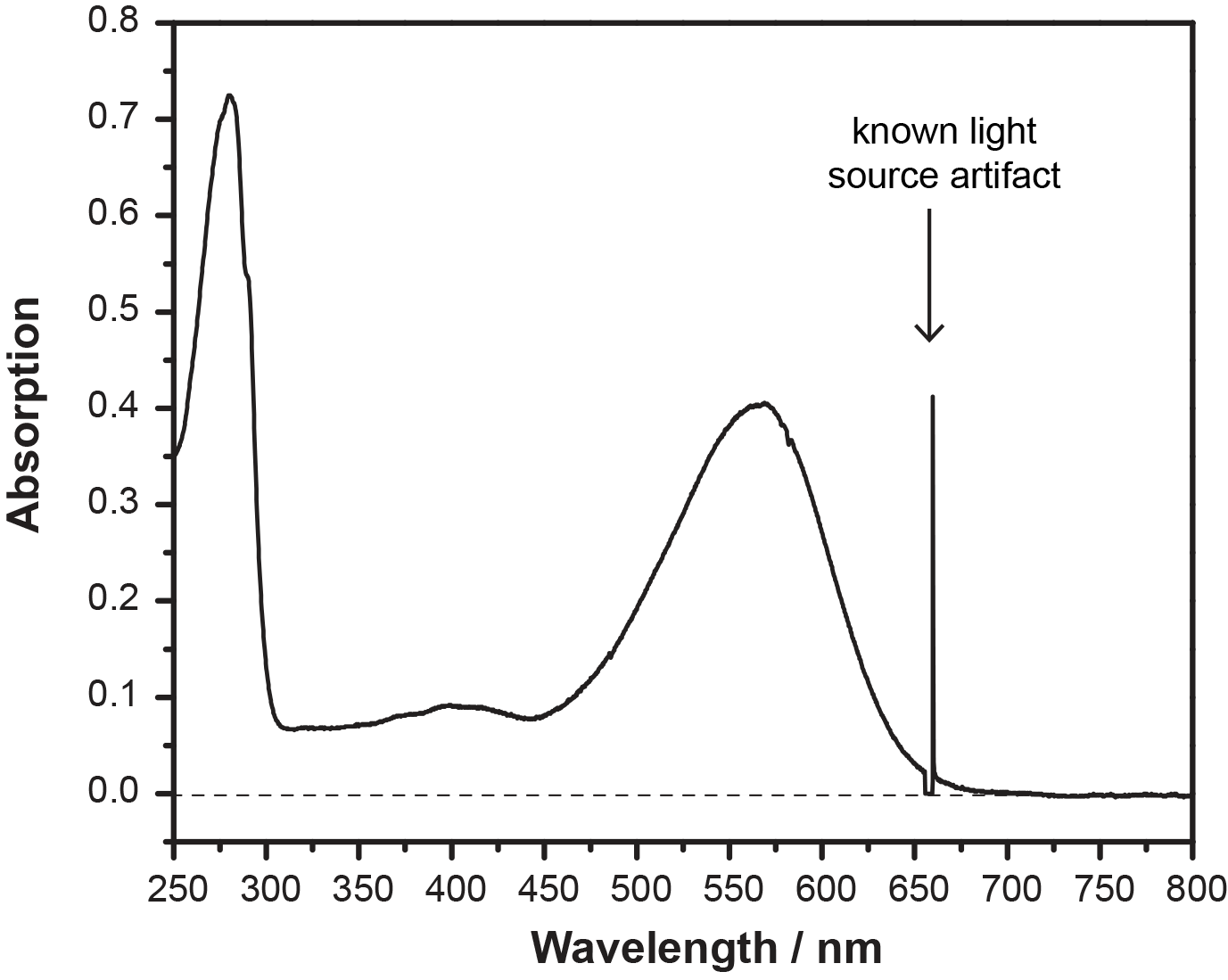


**Figure S6.** Steady state absorption spectrum of PMND at 6.4 μM, from 250 nm to 800 nm. The sample was previously light adapted for more than 45 mins.


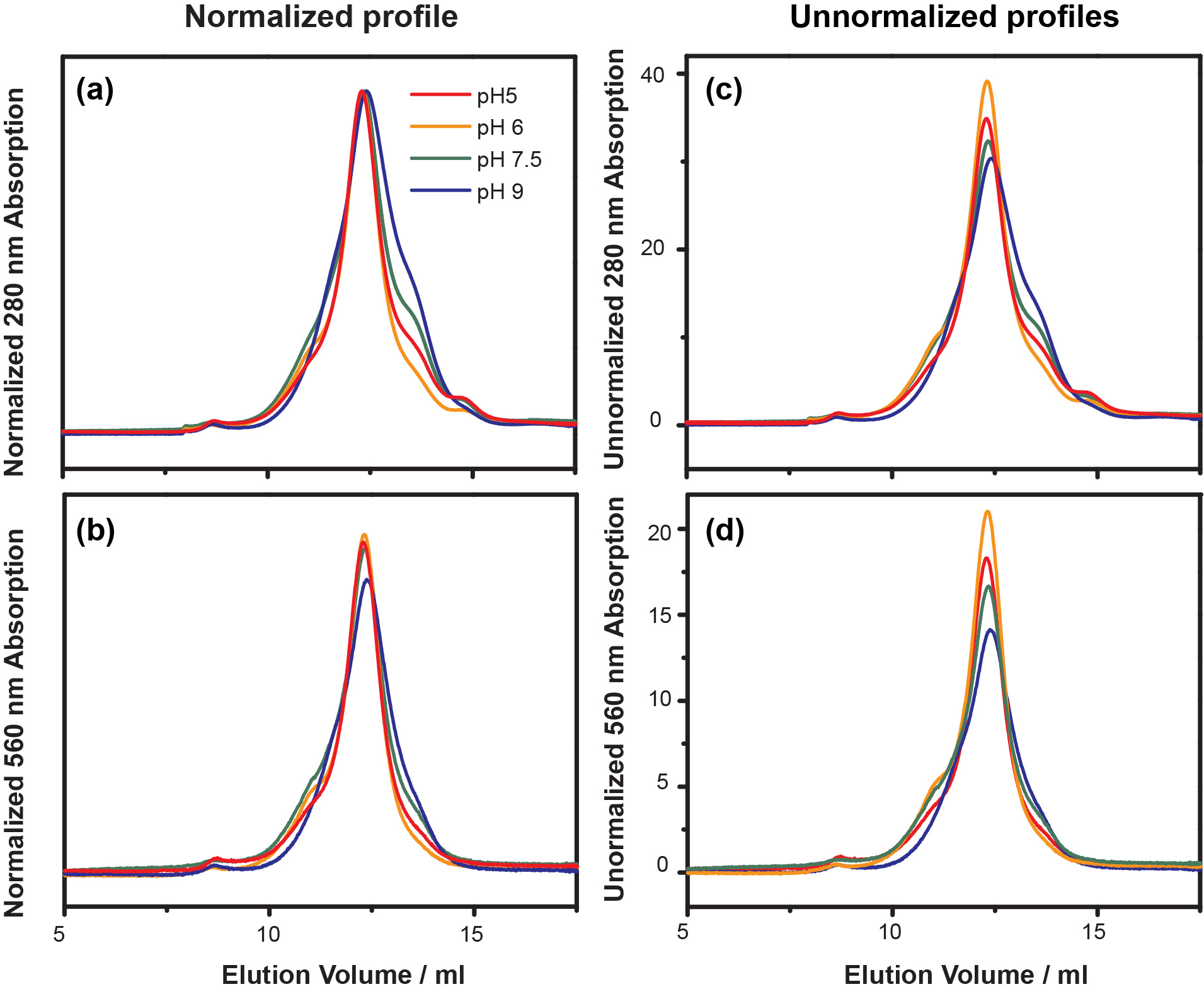


**Figures S7.** (a, b) Normalized and (c, d) unnormalized size exclusion chromatography profiles of PMND assembled at different pHs, monitored at (a, c) 280 nm and (b, d) 560 nm. The normalization (a, b) was achieved by normalizing the profiles monitored at 280 nm. Each experiment was performed with an injection sample of the same volume, and each sample was assembled using the same concentration of protein, NaCl, and respective ratios.
